# A Selected Review of the Mortality Rates of Neonatal Intensive Care Units

**DOI:** 10.3389/fpubh.2015.00225

**Published:** 2015-10-07

**Authors:** Selina Chow, Ronald Chow, Mila Popovic, Michael Lam, Marko Popovic, Joav Merrick, Ruth Naomi Stashefsky Margalit, Henry Lam, Milica Milakovic, Edward Chow, Jelena Popovic

**Affiliations:** ^1^Toronto East General Hospital, Toronto, ON, Canada; ^2^Sunnybrook Health Sciences Centre, Toronto, ON, Canada; ^3^Health Services, Division for Intellectual and Developmental Disabilities, National Institute of Child Health and Human Development, Ministry of Social Affairs, Jerusalem, Israel; ^4^MSR Israel Center for Medical Simulation, Chaim Sheba Medical Center, Tel Hashomer National Education Center, Ramat Gan, Israel

**Keywords:** neonatal intensive care unit, mortality rate, prematurity

## Abstract

**Introduction:**

Newborn babies in need of critical medical attention are normally admitted to the neonatal intensive care unit (NICU). These infants tend to be preterm, have low birth weight, and/or have serious medical conditions. Neonatal survival varies, but progress in perinatal and neonatal care has notably diminished mortality rates. In this selected review, we examine and compare the NICU mortality rates and etiologies of death in different countries.

**Methods:**

A literature search was conducted in Ovid MEDLINE, OLDMEDLINE, EMBASE Classic, and EMBASE. The primary endpoint was the mortality rates in NICUs. Secondary endpoints included the reasons for death and the correlation between infant age and mortality outcome. For the main analysis, we examined all infants admitted to NICUs. Subgroup analyses included extremely low birth weight infants (based on the authors’ own definition), very low birth weight infants, very preterm infants, preterm infants, preterm infants with a birth weight of ≤1,500 g, and by developed and developing countries.

**Results:**

The literature search yielded 1,865 articles, of which 20 were included. The total mortality rates greatly varied among countries. Infants in developed and developing countries had similar ages at death, ranging from 4 to 20 days and 1 to 28.9 days, respectively. The mortality rates ranged from 4 to 46% in developed countries and 0.2 to 64.4% in developing countries.

**Conclusion:**

The mortality rates of NICUs vary between nations but remain high in both developing and developed countries.

## Introduction

Newborn babies in need of critical medical attention are normally admitted to the neonatal intensive care unit (NICU) (1). These infants tend to be preterm (i.e., born before 37 weeks of pregnancy) and have a low birth weight and/or serious medical conditions (1). The combination of advanced technology and a trained medical team (including neonatologists, nurses, respiratory therapists, occupational therapists, dieticians, and lactation consultants) in the NICU effectively provides specialized care for patients (1, 2). Health care professionals are often required to conduct complex assessments, execute highly intensive therapies, and make instant modifications based on the infant’s response in order to intervene before life-threatening conditions emerge (3).

Neonatal survival varies with the quality of medical care (4). Prematurity, congenital anomalies, and perinatal asphyxia are the main causes of death in babies (5). In developed countries, newborns typically die from unpreventable causes, such as congenital abnormalities, whereas the majority of infants in developing countries die from preventable conditions, including infections, birth asphyxia, and prematurity (6). In-hospital mortality rates of NICUs significantly impact infant mortality (7, 8). Infant mortality rates may be stratified into two cohorts by time: neonatal (normally defined as birth – 28 days) and postneonatal (29–364 days) (8). Due to complications from premature births, birth defects, maternal health conditions, difficult labor and delivery, and insufficient access to appropriate care during delivery, approximately two-thirds of all newborn deaths occur in the neonatal period (8). A fraction of NICU deaths in the postneonatal period are attributable to conditions originating from the neonatal period (7).

Progress in perinatal and neonatal care has notably diminished mortality and has improved the health of premature infants admitted to NICUs (9–13). Several publications have reported these findings (14–16), and others have noted the immense variations in mortality rates among NICUs (17, 18). However, no review to date has addressed the similarities and differences between various nations in NICU mortality rates and causes of death. If available, this information could help clinicians, researchers, and policymakers in identifying regions of high need for the provision of limited NICU resources. As such, the purpose of this selected review was to examine and compare the NICU mortality rates and etiologies of death across various countries.

## Materials and Methods

**Figure 1:**
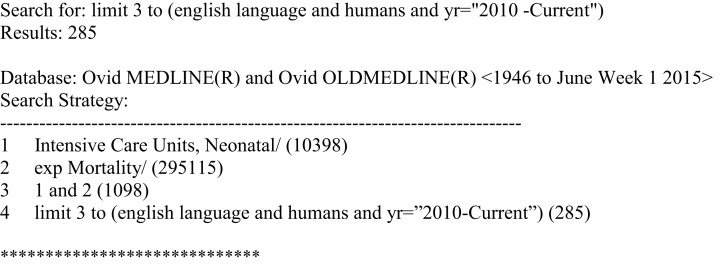
**Search strategy for Ovid MEDLINE and OLDMEDLINE**.

**Figure 2:**
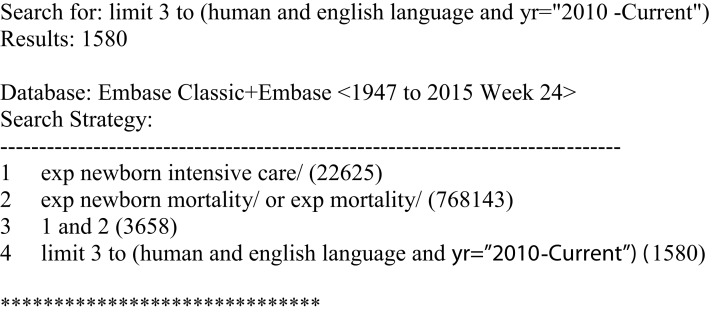
**Search strategy for Embase Classic and Embase**.

A literature search was conducted in Ovid MEDLINE and OLDMEDLINE (1946 to June Week 1, 2015) and EMBASE Classic and EMBASE (1947–2015 Week 24). The key search terms were “neonatal intensive care units” and “mortality” (see Figures 1 and 2). Titles and abstracts were screened to identify if studies were relevant for full-text screening, after which full-texts were included if they met the pre-specified inclusion criteria.

### Selection criteria

Articles were selected for full-text screening if the title or abstract mentioned mortality in addition to either “neonatal intensive care unit” and/or “NICU.” Another requirement was that there had to be a numerical mortality outcome following admittance to the NICU. Articles that did not have mortality rate statistics were excluded. Only English language studies were included. Duplicates of articles found in each database, as well as non-original research and small (i.e., <5 patients) studies, were excluded.

### Data extraction and endpoints

The primary endpoint was the in-hospital mortality rate, which was defined as the number of deceased individuals divided by the sample size. Secondary endpoints included the reasons for death and the correlation between infant age and mortality outcome. For the main analysis, we examined all infants admitted to NICUs. Based on the authors’ own definition, the subgroup analyses were extremely low birth weight infants, very low birth weight infants, very preterm infants, preterm infants, and preterm infants with a birth weight of ≤1,500 g. We also grouped studies based on whether they came from developed or developing countries as classified by the United Nations (19).

## Results

The literature search yielded 1,865 articles, with 285 from MEDLINE and 1,580 from EMBASE. Of those, 40 articles were identified for full-text review as specified by the inclusion criteria; 20 of the 40 articles were rejected after full-text review. Some of the reasons for exclusion were the absence of numerical mortality rates, lack/absence of relevant information regarding NICU mortality, and repeat data. Of the 20 remaining articles (20–39), eight reported on any infants admitted to NICUs (20, 22, 25, 33–37), three reported on the outcomes of extremely low birth weight infants (≤500 g, 401–1,000 g) (21, 26, 38), three others reported on the outcomes of very low birth weight infants (≤1,500 g) (30, 32, 39), two discussed all in-hospital deaths (23, 28), two focused on the outcomes of very preterm infants [<32 weeks gestational age (GA)] (24, 29), one involved the outcomes of preterm infants [<37 weeks GA] (31) and one reported on the outcomes of preterm infants with a birth weight of ≤1,500 g (27) (Table 1).

**Table 1:** **Mortality rates of neonatal intensive care unit in-hospital patients**.

Author	Country	Year(s)	Methods	Age of infants	Mortality outcome	Reasons for death
Feng et al. (20)	Australia	1995–2006	Data for 24,131 infants admitted to 10 NICUs	GA of <24 weeks (*n* = 145) GA of ≥37 weeks (*n* = 6,239) Median age at death: 4 days	Overall mortality rate: 9.2% (2,224/24,131) Mortality rate between 1995 and 2000: 10.3% (1,152/11,185) Mortality rate between 2001 and 2006: 8.3% (1,072/12,946)	Overall leading causes of death: congenital abnormalities (*n* = >445, >20%), respiratory failure (*n* ≈ 400, ~18*%*), HIE (*n* ≈ 222, ~10*%*), infection (*n* ≈ 222, ~10%) Most common causes of death in term infants: congenital abnormalities (40%), HIE (28%) Most common causes of death in preterm infants: respiratory failure (20%) 1995–2000: congenital problems (*n* = 252, 21.9%), respiratory failure (*n* = 217, 18.8%), infection (*n* = 136, 11.8%), HIE (*n* = 128, 11.1%), IVH (*n* = 80, 6.9%), cardiovascular failure (*n* = 56, 4.9%), NEC (*n* = 47, 4.1%), chronic lung disease (*n* = 25, 2.2%), renal failure (*n* = 19, 1.6%), SIDS (*n* = 13, 1.1%), hematological disorders (*n* = 8, 0.7%), neoplasm (*n* = 6, 0.5%), trauma (*n* = 4, 0.3%), maternal conditions (*n* = 3, 0.3%) 2001–2006: congenital problems (*n* = 226, 21.1%), respiratory failure (*n* = 188, 17.5%), HIE (*n* = 129, 12.0%), infection (*n* = 116, 10.8%), IVH (*n* = 112, 10.4%), extreme prematurity (*n* = 76, 7.1%), NEC (*n* = 58, 5.4%), cardiovascular failure (*n* = 36, 3.4%), chronic lung disease (*n* = 21, 2.0%), renal failure (*n* = 20, 1.9%), SIDS (*n* = 14, 1.3%), neoplasm (*n* = 8, 0.7%), maternal conditions (*n* = 6, 0.6%), trauma (*n* = 5, 0.5%), hematological disorders (*n* = 4, 0.4%)
Keir et al. (21)	Australia	2005–2010	Data for 36 extremely low birth weight infants (≤500 g), including 26 NICU patients, born at a tertiary hospital	GA of ≥22 weeks Mean GA of the 12 deceased infants: 24.4 ± 1.4 weeks Median age at death: 20 days	46% (12/26)	NEC (*n* = 2, 17%), fulminating NEC (*n* = 2, 17%), recurrent NEC/multiple cardiorespiratory arrests (*n* = 1, 8%), progressive lactic acidosis – unresponsive to treatment (*n* = 1, 8%), severe lung disease (*n* = 1, 8%), respiratory failure/sepsis (*n* = 1, 8%), massive liver necrosis and fibrosis – etiology unclear (*n* = 1, 8%), severe irrecoverable chronic lung disease (*n* = 1, 8%), lower limb gangrene/cardiorespiratory arrest (*n* = 1, 8%), bilateral grade IV IVH (*n* = 1, 8%)
Sankaran et al. (22)	Canada	January 8, 1996–October 31, 1997	Data for 19,265 infants admitted to 17 tertiary-level NICUs.	Age at death: 2 (*n* = 318, 40%), 3 (*n* = 397, 50%), 12 (*n* = 596, 75%) days since NICU admission; 1 month after admission (*n* = 79, 10%)	Overall mortality rate: 4% (795/19 265) Mortality rate for infants with birth weight ≤500 g: 51% Mortality rate for infants with birth weight ≤1,500 g: 2%	Outborn status (*n* = 334, 42%), GA of <24 weeks (*n* = 310, 39%), chromosomal or congenital anomalies (*n* = 270, 34%), HIE (*n* = 127, 16%), infection (*n* = 111, 14%), GA of 24–28 weeks (*n* = 103, 13%), small for GA (*n* = 79, 10%)
Simpson et al. (23)	Canada	1997, 2002, 2007	Data for 156 in-hospital deaths (53 in 1997, 50 in 2002, 53 in 2007) at a tertiary-level NICU	2007: GA of 23–27 (*n* = 104, 14.3%), 28–32 (*n* = 113, 15.5%), 33–35 (*n* = 102, 14.0%), ≥36 (*n* = 407, 55.9%) weeks Median age at death: 16 days (range: 1–180 days)	Average annual mortality rate between 1988 and 2007: 7.6%	2007: gastrointestinal (*n* = 15, 28.3%), neurologic (*n* = 14, 26.4%), cardiorespiratory disorders (*n* = 10, 18.9%), congenital abnormality (*n* = 8, 15.1%), infection (*n* = 4, 7.5%), extreme prematurity (*n* = 2, 3.8%)
Zhou et al. (24)	China	October 2010–September 2011	Data for 729 very preterm infants admitted to a tertiary NICU	GA of <32 weeks Age at death: 26 (44%) of deaths occurred in the early neonatal period (0–6 days), 28 (49%) in the late neonatal period (7–28 days), 4 (7%) after the neonatal period	Overall mortality rate: 8% (58/729) Mortality rate of extremely low birth weight infants: 27% Mortality rate of very low birth weight infants: 11%	Not listed
Manktelow et al. (25)	England	2008–2010	Data for 2,995 white singleton infants admitted to NICUs in the East Midlands and Yorkshire regions of England	GA of 23 (*n* = 18)–32 weeks (*n* = 1,329)	8.1% (244/2,995)	Not listed
Tagare et al. (26)	India	December 1, 2006–April 30, 2008	Data for 87 extremely low birth weight infants admitted to a level III NICU	Mean GA of deceased infants: 27.2 weeks (range: 26.6–27.8)	45.9% (40/87)	Pulmonary hemorrhage (*n* = 10, 25%), RDS (*n* = 9, 22.5%), IVH (*n* = 9, 22.5%), sepsis (*n* = 8, 20%), NEC (*n* = 1, 2%), BPD (*n* = 1, 1%), pneumothorax (*n* = 1, 1%)
Navaei et al. (27)	Iran	January 2005–March 2006	Data for 194 preterm infants with birth weight of ≤1,500 g who were admitted to two NICUs	GA of 24–27 (*n* = 48), 27–28 (*n* = 74), 28–30 weeks (*n* = 72) Average age at death: 28.9 days	64.4% (125/194)	Prematurity, low birth weight
Eventov-Friedman et al. (28)	Israel	2000–2009	Data for the in-hospital deaths at two tertiary-level NICUs	Age at death: 69 (29%) of the 239 infants died on the first day of life, 31 (13%) dying at up to 48 h of life, 55 (23%) died between days 3 and 7, 53 (22%) died between days 8 and 30, 33 (14%) died after 30 days of life	0.2% (239/96 643)	Overall leading cause of death: prematurity and its complications (*n* = 182, 76%) For infants born at ≤26 weeks: respiratory system failure (due to RDS, air leaks, pulmonary hemorrhage, pulmonary hypertension, BPD) and cardiovascular collapse (57%), ICH (45%), sepsis (18%), NEC (7%) For infants born at ≥37 weeks: congenital anomalies (48%), including cardiac (23%), chromosomal (23%), central nervous system (19%), renal (14%), and lung (9%) anomalies, asphyxia (19%), sepsis (7%) For early deaths (<3 days): severe lung disease in preterm infants (*n* = 60, 60%) For later deaths: sepsis, multiorgan failure For deaths at >30 days of life: chronic lung disease (*n* = 19, 57%), complications of NEC (*n* = 3, 9%)
Corchia et al. (29)	Italy	2005	Data for 4,014 very preterm infants admitted to 105 tertiary-level NICUs	GA of ≤23 (*n* = 167, 4.2%), 24 (*n* = 185, 4.6%), 25 (*n* = 226, 5.6%), 26 (*n* = 285, 7.1%), 27 (*n* = 365, 9.1%), 28 (*n* = 451, 11.2%), 29 (*n* = 560, 14.0%), 30 (*n* = 725, 18.1%), 31 (*n* = 1,048, 26.1%) weeks	18.8% (755/4,014)	Not listed
Shim et al. (30)	Korea	2009	Data for 2,584 very low birth weight infants admitted to NICUs in 76 hospitals	Not listed	Mortality rate for infants with birth weight of <750 g: 44.8% Mortality rate for infants with birth weight of 750–999 g: 20.4% Mortality rate for infants with birth weight of 1,000–1,499 g: 6.5%	Not listed
Shrestha et al. (31)	Nepal	2007–2009	Data for 150 preterm infants admitted to a level III NICU	Mean GA: 30.0 ± 0.37 weeks	Total mortality rate: 20.6% (31/150) Mortality rate in extremely low birth weight infants: 80% (8 deaths) Mortality rate in very low birth weight infants: 39.5% (17 deaths)	HMD (*n* = 20, 64.5%), sepsis (*n* = 18, 58.06%), NEC (*n* = 8, 25.8%), DIC (*n* = 5, 16.1%), pneumonia (*n* = 4, 12.9%), pneumothorax (*n* = 3, 9.6%), neonatal seizure (*n* = 2, 6.45%), shock (*n* = 2, 6.45%), IVH (*n* = 2, 6.45%)
Battin et al. (32)	New Zealand	1959–2009	Data for very low birth weight (≤1,500 g) infants born at a single tertiary neonatal unit	Not listed	Mortality rate of infants with birth weight of 501–1,000 g in 2009: 30% Mortality rate of infants with birth weight of 1,001–1,500 g in 2009: 5%	2008: prematurity and early cardiorespiratory problems (predominantly RDS) (33%), infection (29%), congenital anomalies (12%), NEC (12%)
Ekwochi et al. (33)	Nigeria	June 2012–May 2013	Data for 261 infants admitted to a special care baby unit	Mean age at death: 4.44 days	14.2% (37/261)	Severe form of perinatal asphyxia (*n* = 3, 8%), neonatal sepsis (*n* = 1, 4%)
Costa et al. (34)	Portugal	2004–2008	Data for 1,938 infants admitted to a NICU	Median GA: 34 weeks Median age at death: 10.5 days (range: 0–317 days)	5.7% (110/1,938)	Congenital malformations, including cardiac anomalies (*n* = 55, 50%), prematurity with its complications (*n* = 37, 33.6%), infection (*n* = 5, 4.5%), and HIE (*n* = 3, 2.7%)
Parappil et al. (35)	Qatar	2002–2006	Data for 597 infants admitted to a tertiary-level NICU	GA of 28–32 weeks	Total mortality rate: 6.5% (39/597) Mortality rate for 29 weeks GA: 14.5% (9 deaths) Mortality rate for 30 weeks GA: 12.9% (15 deaths) Mortality rate for 31 weeks GA: 3.9% (6 deaths) Mortality rate for 32 weeks GA: 3.4% (9 deaths) Early mortality rate (<7 days): 35.89% of total mortality (14/39) Late mortality rate (>7 days): 64.11% of total mortality (25/39)	Lethal congenital and chromosomal anomalies (*n* = 12, 30.77%), sepsis (*n* = 11, 28.20%), severe birth asphyxia (*n* = 6, 15.30%), NEC (*n* = 4, 10.25%), pulmonary hemorrhage (*n* = 3, 7.69%), hydrops/congenital infection (*n* = 3, 7.69%)
Pepler et al. (36)	South Africa	2007–2008	Data for 1,578 infants born in 2007 and 2,376 infants born in 2008 admitted to 15 NICUs in private hospitals	Median GA in 2008: 35.9 weeks (range: 23–42.3 weeks)	2007: 3.1% (49/1,578) 2008: 3.8% (91/2,376)	Not listed
Musooko et al. (37)	Uganda	February 1–March 31, 2013	Data for 635 infants, including 341 infants with severe perinatal morbidity, admitted to a NICU	GA of ≥28 weeks Average GA for the 341 infants: 36.0 ± 1.3 weeks Age at death: 17 (45.9%) of the 37 early neonatal deaths occurred on the first day of life, 7 (18.9%) on the second day, 4 (10.8%) on the third day, and 9 (24.3%) between the fourth and seventh days	Overall mortality rate for all NICU patients in 2012: 26–29% Early neonatal death rate (death within 7 days of birth) of the 635 admissions: 12.4% (79/635) Early neonatal death rate of the 341 infants: 10.9% (37/341)	Not listed
Alleman et al. (38)	United States of America	2006–2009	Data for 5,418 extremely low birth weight (401–1,000 g) infants born at 16 Neonatal Research Network centers	GA of 22–28 weeks	Median mortality of all infants in the 16 centers: 34% (~1,842/5,418) (range: 11–53%) Median mortality of infants <25 weeks GA in the 16 centers: 63% (range: 28–90%) Median mortality of infants ≥25 weeks GA in the 16 centers: 16% (range: 7–26%)	Not listed
Lake et al. (39)	United States of America	2007–2008	Data for 72,235 very low birth weight (501–1,500 g) infants born at 558 Vermont Oxford Network hospital NICUs	Mean GA: 28.2 weeks (*n* = 72,235)	12.9% (9,278/71,936)	Not listed

*BPD, bronchopulmonary dysplasia; CDH, congenital diaphragmatic hernia; DIC, disseminated intravascular coagulation; g, grams; GA, gestational age; HIE, hypoxic-ischemic encephalopathy; HMD, hyaline membrane disease; ICH, intracranial hemorrhage; IVH, intraventricular hemorrhage; *n*, number; NEC, necrotizing enterocolitis; NICU, neonatal intensive care unit; RDS, respiratory distress syndrome; SIDS, sudden infant death syndrome; SIP, spontaneous intestinal perforation*.

### Mortality rates in overall admissions to NICUs

Eight studies reported the mortality outcomes for all admissions to the NICU (20, 22, 25, 33–37), five of which examined the age of infants at death (20, 22, 33, 34, 37). Musooko et al. (37) noted that 45.9% (17/37) of infants with early neonatal deaths (death within 7 days of birth) and those with severe perinatal morbidity died on the first day of life in Uganda. The reported median age at death was 4 days in Australia (20), 4.4 days in Nigeria (33), and 10.5 days in Portugal (34). Sankaran et al. (22) found that 75% (596/795) of deceased infants passed away 12 days since NICU admission in Canada (Table 2).

**Table 2:** **Mortality rates of overall admissions to neonatal intensive care units**.

Author	Country	Year(s)	Methods	Age of Infants	Mortality Outcome	Reasons for Death
Feng et al. (20)	Australia	1995–2006	Data for 24,131 infants admitted to 10 NICUs	GA of <24 weeks (*n* = 145) GA of ≥37 weeks (*n* = 6,239) Median age at death: 4 days	Overall mortality rate: 9.2% (2,224/24,131) Mortality rate between 1995 and 2000: 10.3% (1,152/11,185) Mortality rate between 2001 and 2006: 8.3% (1,072/12,946)	Overall leading causes of death: congenital abnormalities (*n* = >445, >20%), respiratory failure (*n* ≈ 400, ~18%), HIE (*n* ≈ 222, ~10%), infection (*n* ≈ 222, ~10%) Most common causes of death in term infants: congenital abnormalities (40%), HIE (28%) Most common causes of death in preterm infants: respiratory failure (20%) 1995–2000: congenital problems (*n* = 252, 21.9%), respiratory failure (*n* = 217, 18.8%), infection (*n* = 136, 11.8%), HIE (*n* = 128, 11.1%), IVH (*n* = 80, 6.9%), cardiovascular failure (*n* = 56, 4.9%), NEC (*n* = 47, 4.1%), chronic lung disease (*n* = 25, 2.2%), renal failure (*n* = 19, 1.6%), SIDS (*n* = 13, 1.1%), hematological disorders (*n* = 8, 0.7%), neoplasm (*n* = 6, 0.5%), trauma (*n* = 4, 0.3%), maternal conditions (*n* = 3, 0.3%) 2001–2006: congenital problems (*n* = 226, 21.1%), respiratory failure (*n* = 188, 17.5%), HIE (*n* = 129, 12.0%), infection (*n* = 116, 10.8%), IVH (*n* = 112, 10.4%), extreme prematurity (*n* = 76, 7.1%), NEC (*n* = 58, 5.4%), cardiovascular failure (*n* = 36, 3.4%), chronic lung disease (*n* = 21, 2.0%), renal failure (*n* = 20, 1.9%), SIDS (*n* = 14, 1.3%), neoplasm (*n* = 8, 0.7%), maternal conditions (*n* = 6, 0.6%), trauma (*n* = 5, 0.5%), hematological disorders (*n* = 4, 0.4%)
Sankaran et al. (22)	Canada	January 8, 1996–October 31, 1997	Data for 19,265 infants admitted to 17 tertiary-level NICUs	Age at death: 2 (*n* = 318, 40%), 3 (*n* = 397, 50%), 12 (*n* = 596, 75%) days since NICU admission; 1 month after admission (*n* = 79, 10%)	Overall mortality rate: 4% (795/19,265) Mortality rate for infants with birth weight <500 g: 51% Mortality rate for infants with birth weight ≤1,500 g: 2%	Outborn status (*n* = 334, 42%), GA of <24 weeks (*n* = 310, 39%), chromosomal or congenital anomalies (*n* = 270, 34%), HIE (*n* = 127, 16%), infection (*n* = 111, 14%), GA of 24–28 weeks (*n* = 103, 13%), small for GA (*n* = 79, 10%)
Manktelow et al. (25)	England	2008–2010	Data for 2,995 white singleton infants admitted to NICUs in the East Midlands and Yorkshire regions of England	GA of 23 (*n* = 18)–32 weeks (*n* = 1,329)	8.1% (244/2,995)	Not listed
Ekwochi et al. (33)	Nigeria	June 2012–May 2013	Data for 261 infants admitted to a special care baby unit	Mean age at death: 4.44 days	14.2% (37/261)	Severe form of perinatal asphyxia (*n* = 3, 8%), neonatal sepsis (*n* = 1, 4%)
Costa et al. (34)	Portugal	2004–2008	Data for 1,938 infants admitted to a NICU	Median GA: 34 weeks Median age at death: 10.5 days (range: 0–317 days)	5.7% (110/1,938)	Congenital malformations, including cardiac anomalies (*n* = 55, 50%), prematurity with its complications (*n* = 37, 33.6%), infection (*n* = 5, 4.5%), and HIE (*n* = 3, 2.7%)
Parappil et al. (35)	Qatar	2002–2006	Data for 597 infants admitted to a tertiary-level NICU	GA of 28–32 weeks	Total mortality rate: 6.5% (39/597) Mortality rate for 29 weeks GA: 14.5% (nine deaths) Mortality rate for 30 weeks GA: 12.9% (15 deaths) Mortality rate for 31 weeks GA: 3.9% (six deaths) Mortality rate for 32 weeks GA: 3.4% (nine deaths) Early mortality rate (<7 days): 35.89% of total mortality (14/39) Late mortality rate (>7 days): 64.11% of total mortality (25/39)	Lethal congenital and chromosomal anomalies (*n* = 12, 30.77%), sepsis (*n* = 11, 28.20%), severe birth asphyxia (*n* = 6, 15.30%), NEC (*n* = 4, 10.25%), pulmonary hemorrhage (*n* = 3, 7.69%), hydrops/congenital infection (*n* = 3, 7.69%)
Pepler et al. (36)	South Africa	2007–2008	Data for 1,578 infants born in 2007 and 2,376 infants born in 2008 admitted to 15 NICUs in private hospitals	Median GA in 2008: 35.9 weeks (range: 23–42.3 weeks)	2007: 3.1% (49/1,578) 2008: 3.8% (91/2,376)	Not listed
Musooko et al. (37)	Uganda	February 1–March 31, 2013	Data for 635 infants, including 341 infants with severe perinatal morbidity, admitted to a NICU	GA of ≥28 weeks Average GA for the 341 infants: 36.0 ± 1.3 weeks Age at death: 17 (45.9%) of the 37 early neonatal deaths occurred on the first day of life, 7 (18.9%) on the second day, 4 (10.8%) on the third day, and 9 (24.3%) between the fourth and seventh days	Overall mortality rate for all NICU patients in 2012: 26–29% Early neonatal death rate (death within 7 days of birth) of the 635 admissions: 12.4% (79/635) Early neonatal death rate of the 341 infants: 10.9% (37/341)	Not listed

*g, grams; GA, gestational age; HIE, hypoxic-ischemic encephalopathy; IVH, intraventricular hemorrhage; *n*, number; NEC, necrotizing enterocolitis; NICU, neonatal intensive care unit; SIDS, sudden infant death syndrome*.

The total mortality rates varied between countries. The overall in-hospital mortality rates were reported to be 3.1 and 3.8% in South Africa in 2007 and 2008, respectively (36), 4% in Canada from January 1996 to October 1997 (22), 5.7% in Portugal between 2004 and 2008 (34), 6.5% in Qatar from 2002 to 2006 (35), 8.1% in England between 2008 and 2010 (25), 9.2% in Australia across 1995–2006 (20), 14.2% in Nigeria between June 2012 and May 2013 (33), and 26–29% in Uganda in 2012 (37).

Five publications (20, 22, 33–35) investigated the etiologies of death in NICUs. Congenital and/or chromosomal abnormalities/malformations were common causes of death in Australia [greater than 20% (445/2,224)] (20), Canada [34% (270/795)] (22), Portugal [50% (55/110)] (34), and Qatar [30.77% (12/39)] (35). The common pathologies that led to death were hypoxic-ischemic encephalopathy (HIE) and infection. Though not as prevalent as congenital abnormalities, HIE and infection were still reported as common causes of death in Australia (10% (222/2,224) for both) (20), Canada [HIE = 16% (127/795); infection = 14% (111/795)] (22), Portugal [HIE = 2.7% (3/111); infection = 4.5% (5/110)] (34), and Qatar [hydrops/congenital infection = 7.69% (3/39)] (35).

Severe forms of perinatal asphyxia and neonatal sepsis were among the primary reasons for death in Nigeria [asphyxia = 8% (3/37); sepsis = 4% (1/37)] (33) and Qatar [asphyxia = 15.30% (6/39); sepsis = 28.20% (11/39)] (35). Other common causes of death in the NICU included respiratory failure in Australia [18% (400/2,224)] (20), outborn status in Canada [42% (334/795)] (22), GA less than 24 weeks in Canada [39% (310/795)] (22), GA between 24 and 28 weeks in Canada [13% (103/795)] (22), small size and/or weight for GA in Canada [10% (79/795)] (22), very low birth weight in Nigeria [0.8% (0.3/37)] (33), complications of prematurity in Portugal [33.6% (37/110)] (34), necrotizing enterocolitis (NEC) in Qatar [10.25% (4/39)] (35), and pulmonary hemorrhage in Qatar [7.69% (3/39)] (35).

### Mortality rates in extremely low birth weight infants (≤500 g, 401–1,000 g)

Three studies reported the mortality outcomes for extremely low birth weight infants (21, 26, 38). Each study had its own definition of extremely low birth weight infants. For instance, Keir et al. (21) defined extremely low birth weight as ≤500 g, while Alleman et al. (38) defined extremely low birth weight as 401–1,000 g.

Two articles (21, 26) studied the mean age of infants with extremely low birth weights. Keir et al. (21) found that the mean GA of the deceased infants was 24.4 ± 1.4 weeks in Australia, while Tagare et al. (26) noted that the mean GA of the late infants was 27.2 weeks in India (Table 3). Mortality rates were reported to be 34% in United States between 2006 and 2009 (38), 45.9% in India between December 2006 and April 2008 (26), and 46% in Australia between 2005 and 2010 (21).

**Table 3:** **Mortality rates of extremely low birth weight infants (≤500 g, 401–1,000 g)**.

Author	Country	Year(s)	Methods	Age of infants	Mortality outcome	Reasons for death
Keir et al. (21)	Australia	2005–2010	Data for 36 extremely low birth weight infants (≤500 g), including 26 NICU patients, born at a tertiary hospital	GA of ≥22 weeks Mean GA of the 12 deceased infants: 24.4 ± 1.4 weeks Median age at death: 20 days	46% (12/26)	NEC (*n* = 2, 17%), fulminating NEC (*n* = 2, 17%), recurrent NEC/multiple cardiorespiratory arrests (*n* = 1, 8%), progressive lactic acidosis – unresponsive to treatment (*n* = 1, 8%), severe lung disease (*n* = 1, 8%), respiratory failure/sepsis (*n* = 1, 8%), massive liver necrosis and fibrosis – etiology unclear (*n* = 1, 8%), severe irrecoverable chronic lung disease (*n* = 1, 8%), lower limb gangrene/cardiorespiratory arrest (*n* = 1, 8%), bilateral grade IV IVH (*n* = 1, 8%)
Tagare et al. (26)	India	December 1, 2006–April 30, 2008	Data for 87 extremely low birth weight infants admitted to a level III NICU	Mean GA of deceased infants: 27.2 weeks (range: 26.6–27.8)	45.9% (40/87)	Pulmonary hemorrhage (*n* = 10, 25%), RDS (*n* = 9, 22.5%), IVH (*n* = 9, 22.5%), sepsis (*n* = 8, 20%), NEC (*n* = 1, 2%), BPD (*n* = 1, 1%), pneumothorax (*n* = 1, 1%)
Alleman et al. (38)	United States of America	2006–2009	Data for 5,418 extremely low birth weight (401–1,000 g) infants born at 16 Neonatal Research Network centers	GA of 22–28 weeks	Median mortality of all infants in the 16 centers: 34% (~1,842/5,418) (range: 11–53%) Median mortality of infants <25 weeks GA in the 16 centers: 63% (range: 28–90%) Median mortality of infants ≥25 weeks GA in the 16 centers: 16% (range: 7–26%)	Not listed

*BPD, bronchopulmonary dysplasia; g, grams; GA, gestational age; IVH, intraventricular hemorrhage; *N*, number; NEC, necrotizing enterocolitis; NICU, neonatal intensive care unit; RDS, respiratory distress syndrome*.

Two publications (21, 26) investigated the etiologies of death of extremely low birth weight infants. In Australia, NEC [17% (2/12)] and fulminating NEC [17% (2/12)] were the two most common causes of death (21). In India, the leading reasons of death were pulmonary hemorrhage [25% (10/40)], respiratory distress syndrome [RDS, 22.5% (9/40)], intraventricular hemorrhage [IVH, 22.5% (9/40)], and sepsis [20% (8/40)] (26).

### Mortality rates in very low birth weight infants (≤1,500 g)

Three studies reported the mortality outcomes for very low birth weight infants (30, 32, 39). The overall in-hospital mortality rates were reported to be 5% in New Zealand for infants with birth weight of 1,001–1,500 g in 2009 (32), 6.5% in Korea for infants with birth weight of 1,000–1,499 g in 2009 (30), 12.9% in United States of America between 2007 and 2008 (39), 20.4% in Korea for infants with birth weight of 750–999 g in 2009 (30), 30% in New Zealand for infants with birth weight of 501–1,000 g in 2009 (32), and 44.8% in Korea for infants with birth weight of <750 g in 2009 (30) (Table 4).

**Table 4:** **Mortality rates of very low birth weight infants (≤1,500 g)**.

Author	Country	Year(s)	Methods	Age of infants	Mortality outcome	Reasons for death
Shim et al. (30)	Korea	2009	Data for 2,584 very low birth weight infants admitted to NICUs in 76 hospitals	Not listed	Mortality rate for infants with birth weight of <750 g: 44.8% Mortality rate for infants with birth weight of 750–999 g: 20.4% Mortality rate for infants with birth weight of 1,000–1,499 g: 6.5%	Not listed
Battin et al. (32)	New Zealand	1959–2009	Data for very low birth weight (≤1,500 g) infants born at a single tertiary neonatal unit	Not listed	Mortality rate of infants with birth weight of 501–1,000 g in 2009: 30% Mortality rate of infants with birth weight of 1,001–1,500 g in 2009: 5%	2008: prematurity and early cardiorespiratory problems (predominantly RDS) (33%), infection (29%), congenital anomalies (12%), NEC (12%)
Lake et al. (39)	United States of America	2007–2008	Data for 72,235 very low birth weight (501–1,500 g) infants born at 558 Vermont Oxford Network hospital NICUs	Mean GA: 28.2 weeks (*n* = 72,235)	12.9% (9,278/71,936)	Not listed

*g, grams; GA, gestational age; *n*, number; NEC, necrotizing enterocolitis; NICU, neonatal intensive care unit; RDS, respiratory distress syndrome*.

Battin et al. (32) investigated the etiologies of death in infants with very low birth weights. During 2008, in a NICU in New Zealand, prematurity and early cardiorespiratory problems (predominantly RDS) (33%), infection (29%), congenital anomalies (12%), and NEC (12%) were the leading causes of death (32).

### Mortality rates in overall in-hospital deaths

Two studies reported on all in-hospital deaths at a NICU, for which both trials defined the in-hospital mortality as all inpatient deaths during the study period (23, 28). However, each study calculated the in-hospital mortality rate differently; Simpson et al. (23) computed the figure per 100 admissions, while Eventov-Friedman et al. (28) did it per 1,000 live births. Simpson et al. (23) reported that the median age at death was 16 days in Canada (23), and Eventov-Friedman et al. (28) noted that 29% (69/239) of deceased infants died on the first day of life in Israel. The overall in-hospital mortality rates were reported as 0.2% in Israel between 2000 and 2009 (28), and 7.6% in Canada between 1988 and 2007 (23). Prematurity and its complications was a common cause of death; in Israel, 76% (182/239) of late infants died from these circumstances (28), but only 3.8% (2/53) of perished neonates died from extreme prematurity in Canada (23). The other leading reasons of death in Canada were gastrointestinal disorders [28.3% (15/53)], neurologic disorders [(26.4% (14/53)], cardiorespiratory disorders [18.9% (10/53)], congenital abnormalities [15.1% (8/53)], and infection [7.5% (4/53)] (23) (Table 5).

**Table 5:** **Mortality rates of overall in-hospital patients**.

Author	Country	Year(s)	Methods	Age of infants	Mortality outcome	Reasons for death
Simpson et al. (23)	Canada	1997, 2002, 2007	Data for 156 in-hospital deaths (53 in 1997, 50 in 2002, 53 in 2007) at a tertiary-level NICU	2007: GA of 23–27 (*n* = 104, 14.3%), 28–32 (*n* = 113, 15.5%), 33–35 (*n* = 102, 14.0%), ≥36 (*n* = 407, 55.9%) weeks Median age at death: 16 days (range: 1–180 days)	Average annual mortality rate between 1988 and 2007: 7.6%	2007: gastrointestinal (*n* = 15, 28.3%), neurologic (*n* = 14, 26.4%), cardiorespiratory disorders (*n* = 10, 18.9%), congenital abnormality (*n* = 8, 15.1%), infection (*n* = 4, 7.5%), extreme prematurity (*n* = 2, 3.8%)
Eventov-Friedman et al. (28)	Israel	2000–2009	Data for the in-hospital deaths at two tertiary-level NICUs	Age at death: 69 (29%) of the 239 infants died on the first day of life, 31 (13%) dying at up to 48 h of life, 55 (23%) died between days 3 and 7, 53 (22%) died between days 8 and 30, 33 (14%) died after 30 days of life	0.2% (239/96 643)	Overall leading cause of death: prematurity and its complications (*n* = 182, 76%) For infants born at ≤26 weeks: respiratory system failure (due to RDS, air leaks, pulmonary hemorrhage, pulmonary hypertension, BPD) and cardiovascular collapse (57%), ICH (45%), sepsis (18%), NEC (7%) For infants born at ≥37 weeks: congenital anomalies (48%), including cardiac (23%), chromosomal (23%), central nervous system (19%), renal (14%), and lung (9%) anomalies, asphyxia (19%), sepsis (7%) For early deaths (<3 days): severe lung disease in preterm infants (*n* = 60, 60%) For later deaths: sepsis, multiorgan failure For deaths at >30 days of life: chronic lung disease (*n* = 19, 57%), complications of NEC (*n* = 3, 9%)

*BPD, bronchopulmonary dysplasia; GA, gestational age; ICH, intracranial hemorrhage; *n*, number; NEC, necrotizing enterocolitis; NICU, neonatal intensive care unit; RDS, respiratory distress syndrome*.

### Mortality rates in very preterm infants (<32 weeks GA)

Two studies reported the mortality outcomes for very preterm infants (24, 29). Zhou et al. (24) examined the age at death, noting that 49% (28/58) of deceased infants died in the late neonatal period (7–28 days). The overall in-hospital mortality rates were noted to be 8% in China between October 2010 and September 2011 (24), and 18.8% in Italy in 2005 (29) (Table 6).

**Table 6:** **Mortality rates of very preterm infants (<32 weeks gestational age)**.

Author	Country	Year(s)	Methods	Age of infants	Mortality outcome	Reasons for death
Zhou et al. (24)	China	October 2010–September 2011	Data for 729 very preterm infants admitted to a tertiary NICU	GA of <32 weeks Age at death: 26 (44%) of deaths occurred in the early neonatal period (0–6 days), 28 (49%) in the late neonatal period (7–28 days), 4 (7%) after the neonatal period	Overall mortality rate: 8% (58/729) Mortality rate of extremely low birth weight infants: 27% Mortality rate of very low birth weight infants: 11%	Not listed
Corchia et al. (29)	Italy	2005	Data for 4,014 very preterm infants admitted to 105 tertiary-level NICUs	GA of ≤23 (*n* = 167, 4.2%), 24 (*n* = 185, 4.6%), 25 (*n* = 226, 5.6%), 26 (*n* = 285, 7.1%), 27 (*n* = 365, 9.1%), 28 (*n* = 451, 11.2%), 29 (*n* = 560, 14.0%), 30 (*n* = 725, 18.1%), 31 (*n* = 1,048, 26.1%) weeks	18.8% (755/4,014)	Not listed

*GA, gestational age; *n*, number*.

### Mortality rates in preterm infants (<37 weeks GA)

One study reported the mortality outcomes for preterm infants (31). The overall in-hospital mortality rate was 20.6% in Nepal between 2007 and 2009 (31) (Table 7). Additionally, the primary etiologies of death were hyaline membrane disease [HMD, 64.5% (20/31)], sepsis [58.06% (18/31)], and NEC [25.8% (8/31)] (31).

**Table 7:** **Mortality rates of preterm infants (<37 weeks gestational age)**.

Author	Country	Year(s)	Methods	Age of infants	Mortality outcome	Reasons for death
Shrestha et al. (31)	Nepal	2007–2009	Data for 150 preterm infants admitted to a level III NICU	Mean GA: 30.0 ± 0.37 weeks	Total mortality rate: 20.6% (31/150) Mortality rate in extremely low birth weight infants: 80% (eight deaths) Mortality rate in very low birth weight infants: 39.5% (17 deaths)	HMD (*n* = 20, 64.5%), sepsis (*n* = 18, 58.06%), NEC (*n* = 8, 25.8%), DIC (*n* = 5, 16.1%), pneumonia (*n* = 4, 12.9%), pneumothorax (*n* = 3, 9.6%), neonatal seizure (*n* = 2, 6.45%), shock (*n* = 2, 6.45%), IVH (*n* = 2, 6.45%)

*DIC, disseminated intravascular coagulation; GA, gestational age; HMD, hyaline membrane disease; IVH, intraventricular hemorrhage; *n*, number; NEC, necrotizing enterocolitis; NICU, neonatal intensive care unit*.

### Age of death and mortality rates of developed and developing countries

Of the 20 articles included in this selected review (20–39), 10 reported on the NICU mortality outcomes in developed countries (20–23, 25, 29, 32, 34, 38, 39) and 10 reported on those of developing countries (24, 26–28, 30, 31, 33, 35–37).

Infants in developed and developing countries had similar ages at death, ranging from 4 to 20 days (20–23, 34) (Table 8) and 1 to 28.9 days (24, 27, 28, 33, 37) (Table 9), respectively. The mortality rates ranged from 4 to 46% in developed countries (20–23, 25, 29, 32, 34, 38, 39) and 0.2 to 64.4% in developing countries (24, 26–28, 30, 31, 33, 35–37).

**Table 8:** **Mortality rates of patients from developed countries**.

Author	Country	Year(s)	Methods	Age of infants	Mortality outcome	Reasons for death
Feng et al. (20)	Australia	1995–2006	Data for 24,131 infants admitted to 10 NICUs	GA <24 weeks (*n* = 145) GA ≥37 weeks (*n* = 6,239) Median age at death: 4 days	Overall mortality rate: 9.2% (2,224/24,131) Mortality rate between 1995 and 2000: 10.3% (1,152/11,185) Mortality rate between 2001 and 2006: 8.3% (1,072/12,946)	Overall leading causes of death: congenital abnormalities (*n* = >445, >20%), respiratory failure (*n* ≈ 400, ~ 18%), HIE (*n* ≈ 222, ~ 10%), infection (*n* ≈ 222, ~ 10%) Most common causes of death in term infants: congenital abnormalities (40%), HIE (28%) Most common causes of death in preterm infants: respiratory failure (20%) 1995–2000: congenital problems (*n* = 252, 21.9%), respiratory failure (*n* = 217, 18.8%), infection (*n* = 136, 11.8%), HIE (*n* = 128, 11.1%), IVH (*n* = 80, 6.9%), cardiovascular failure (*n* = 56, 4.9%), NEC (*n* = 47, 4.1%), chronic lung disease (*n* = 25, 2.2%), renal failure (*n* = 19, 1.6%), SIDS (*n* = 13, 1.1%), hematological disorders (*n* = 8, 0.7%), neoplasm (*n* = 6, 0.5%), trauma (*n* = 4, 0.3%), maternal conditions (*n* = 3, 0.3%) 2001–2006: congenital problems (*n* = 226, 21.1%), respiratory failure (*n* = 188, 17.5%), HIE (*n* = 129, 12.0%), infection (*n* = 116, 10.8%), IVH (*n* = 112, 10.4%), extreme prematurity (*n* = 76, 7.1%), NEC (*n* = 58, 5.4%), cardiovascular failure (*n* = 36, 3.4%), chronic lung disease (*n* = 21, 2.0%), renal failure (*n* = 20, 1.9%), SIDS (*n* = 14, 1.3%), neoplasm (*n* = 8, 0.7%), maternal conditions (*n* = 6, 0.6%), trauma (*n* = 5, 0.5%), hematological disorders (*n* = 4, 0.4%)
Keir et al. (21)	Australia	2005–2010	Data for 36 extremely low birth weight infants (≤500 g), including 26 NICU patients, born at a tertiary hospital	GA of ≥22 weeks Mean GA of the 12 deceased infants: 24.4 ± 1.4 weeks Median age at death: 20 days	46% (12/26)	NEC (*n* = 2, 17%), fulminating NEC (*n* = 2, 17%), recurrent NEC/multiple cardiorespiratory arrests (*n* = 1, 8%), progressive lactic acidosis – unresponsive to treatment (*n* = 1, 8%), severe lung disease (*n* = 1, 8%), respiratory failure/sepsis (*n* = 1, 8%), massive liver necrosis and fibrosis – etiology unclear (*n* = 1, 8%), severe irrecoverable chronic lung disease (*n* = 1, 8%), lower limb gangrene/cardiorespiratory arrest (*n* = 1, 8%), bilateral grade IV IVH (*n* = 1, 8%)
Sankaran et al. (22)	Canada	January 8, 1996–October 31, 1997	Data for 19,265 infants admitted to 17 tertiary-level NICUs	Age at death: 2 (*n* = 318, 40%), 3 (*n* = 397, 50%), 12 (*n* = 596, 75%) days since NICU admission; 1 month after admission (*n* = 79, 10%)	Overall mortality rate: 4% (795/19,265) Mortality rate for infants with birth weight <500 g: 51% Mortality rate for infants with birth weight ≤1,500 g: 2%	Outborn status (*n* = 334, 42%), GA < 24 weeks (*n* = 310, 39%), chromosomal or congenital anomalies (*n* = 270, 34%), HIE (*n* = 127, 16%), infection (*n* = 111, 14%), GA 24–28 weeks (*n* = 103, 13%), small for GA (*n* = 79, 10%)
Simpson et al. (23)	Canada	1997, 2002, 2007	Data for 156 in-hospital deaths (53 in 1997, 50 in 2002, 53 in 2007) at a tertiary-level NICU	2007: GA of 23–27 (*n* = 104, 14.3%), 28–32 (*n* = 113, 15.5%), 33–35 (*n* = 102, 14.0%), ≥36 (*n* = 407, 55.9%) weeks Median age at death: 16 days (range: 1–180 days)	Average annual mortality rate between 1988 and 2007: 7.6%	2007: gastrointestinal (*n* = 15, 28.3%), neurologic (*n* = 14, 26.4%), cardiorespiratory disorders (*n* = 10, 18.9%), congenital abnormality (*n* = 8, 15.1%), infection (*n* = 4, 7.5%), extreme prematurity (*n* = 2, 3.8%)
Manktelow et al. (25)	England	2008–2010	Data for 2,995 white singleton infants admitted to NICUs in the East Midlands and Yorkshire regions of England	GA of 23 (*n* = 18)–32 weeks (*n* = 1,329)	8.1% (244/2,995)	Not listed
Corchia et al. (29)	Italy	2005	Data for 4,014 very preterm infants admitted to 105 tertiary-level NICUs	GA of ≤ 23 (*n* = 167, 4.2%), 24 (*n* = 185, 4.6%), 25 (*n* = 226, 5.6%), 26 (*n* = 285, 7.1%), 27 (*n* = 365, 9.1%), 28 (*n* = 451, 11.2%), 29 (*n* = 560, 14.0%), 30 (*n* = 725, 18.1%), 31 (*n* = 1,048, 26.1%) weeks	18.8% (755/4,014)	Not listed
Battin et al. (32)	New Zealand	1959–2009	Data for very low birth weight (≤1,500 g) infants born at a single tertiary neonatal unit	Not listed	Mortality rate of infants with birth weight of 501–1,000 g in 2009: 30% Mortality rate of infants with birth weight of 1,001– 1,500 g in 2009: 5%	2008: prematurity and early cardiorespiratory problems (predominantly RDS) (33%), infection (29%), congenital anomalies (12%), NEC (12%)
Costa et al. (34)	Portugal	2004–2008	Data for 1,938 infants admitted to a NICU	Median GA: 34 weeks Median age at death: 10.5 days (range: 0–317 days)	5.7% (110/1,938)	Congenital malformations, including cardiac anomalies (*n* = 55, 50%), prematurity with its complications (*n* = 37, 33.6%), infection (*n* = 5, 4.5%), and HIE (*n* = 3, 2.7%)
Alleman et al. (38)	United States of America	2006–2009	Data for 5,418 extremely low birth weight (401–1,000 g) infants born at 16 Neonatal Research Network centers	GA of 22–28 weeks	Median mortality of all infants in the 16 centers: 34% (~1,842/5,418) (range: 11–53%)Median mortality of infants <25 weeks GA in the 16 centers: 63% (range: 28–90%) Median mortality of infants ≥25 weeks GA in the 16 centers: 16% (range: 7–26%)	Not listed
Lake et al. (39)	United States of America	2007–2008	Data for 72,235 very low birth weight (501–1,500 g) infants born at 558 Vermont Oxford Network hospital NICUs	Mean GA: 28.2 weeks (*n* = 72,235)	12.9% (9,278/71,936)	Not listed

*g, grams; GA, gestational age; HIE, hypoxic-ischemic encephalopathy; IVH, intraventricular hemorrhage; *n*, number; NEC, necrotizing enterocolitis; NICU, neonatal intensive care unit; RDS, respiratory distress syndrome; SIDS, sudden infant death syndrome*.

**Table 9:** **Mortality rates of patients from developing countries**.

Author	Country	Year(s)	Methods	Age of infants	Mortality outcome	Reasons for death
Zhou et al. (24)	China	October 2010–September 2011	Data for 729 very preterm infants admitted to a tertiary NICU	GA of <32 weeks Age at death: 26 (44%) of deaths occurred in the early neonatal period (0–6 days), 28 (49%) in the late neonatal period (7–28 days), 4 (7%) after the neonatal period	Overall mortality rate: 8% (58/729) Mortality rate of extremely low birth weight infants: 27% Mortality rate of very low birth weight infants: 11%	Not listed
Tagare et al. (26)	India	December 1, 2006–April 30, 2008	Data for 87 extremely low birth weight infants admitted to a level III NICU.	Mean GA of deceased infants: 27.2 weeks (range: 26.6–27.8)	45.9% (40/87)	Pulmonary hemorrhage (*n* = 10, 25%), RDS (*n* = 9, 22.5%), IVH (*n* = 9, 22.5%), sepsis (*n* = 8, 20%), NEC (*n* = 1, 2%), BPD (*n* = 1, 1%), pneumothorax (*n* = 1, 1%)
Navaei et al. (27)	Iran	January 2005–March 2006	Data for 194 preterm infants with birth weight of ≤1,500 g who were admitted to 2 NICUs	GA of 24–27 (*n* = 48), 27–28 (*n* = 74), 28–30 weeks (*n* = 72)Average age at death: 28.9 days	64.4% (125/194)	Prematurity, low birth weight
Eventov-Friedman et al. (28)	Israel	2000–2009	Data for the in-hospital deaths at two tertiary-level NICUs	Age at death: 69 (29%) of the 239 infants died on the first day of life, 31 (13%) dying at up to 48 h of life, 55 (23%) died between days 3 and 7, 53 (22%) died between days 8 and 30, 33 (14%) died after 30 days of life	0.2% (239/96,643)	Overall leading cause of death: prematurity and its complications (*n* = 182, 76%) For infants born at ≤26 weeks: respiratory system failure (due to RDS, air leaks, pulmonary hemorrhage, pulmonary hypertension, BPD) and cardiovascular collapse (57%), ICH (45%), sepsis (18%), NEC (7%) For infants born at ≥37 weeks: congenital anomalies (48%), including cardiac (23%), chromosomal (23%), central nervous system (19%), renal (14%), and lung (9%) anomalies, asphyxia (19%), sepsis (7%) For early deaths (<3 days): severe lung disease in preterm infants (*n* = 60, 60%) For later deaths: sepsis, multiorgan failure For deaths at >30 days of life: chronic lung disease (*n* = 19, 57%), complications of NEC (*n* = 3, 9%)
Shim et al. (30)	Korea	2009	Data for 2,584 very low birth weight infants admitted to NICUs in 76 hospitals	Not listed	Mortality rate for infants with birth weight of < 750 g: 44.8% Mortality rate for infants with birth weight of 750–999 g: 20.4% Mortality rate for infants with birth weight of 1,000–1,499 g: 6.5%	Not listed
Shrestha et al. (31)	Nepal	2007–2009	Data for 150 preterm infants admitted to a level III NICU	Mean GA: 30.0 ± 0.37 weeks	Total mortality rate: 20.6% (31/150) Mortality rate in extremely low birth weight infants: 80% (eight deaths) Mortality rate in very low birth weight infants: 39.5% (17 deaths)	HMD (*n* = 20, 64.5%), sepsis (*n* = 18, 58.06%), NEC (*n* = 8, 25.8%), DIC (*n* = 5, 16.1%), pneumonia (*n* = 4, 12.9%), pneumothorax (*n* = 3, 9.6%), neonatal seizure (*n* = 2, 6.45%), shock (*n* = 2, 6.45%), IVH (*n* = 2, 6.45%)
Ekwochi et al. (33)	Nigeria	June 2012–May 2013	Data for 261 infants admitted to a special care baby unit	Mean age at death: 4.44 days	14.2% (37/261)	Severe form of perinatal asphyxia (*n* = 3, 8%), neonatal sepsis (*n* = 1, 4%)
Parappil et al. (35)	Qatar	2002–2006	Data for 597 infants admitted to a tertiary-level NICU	GA of 28–32 weeks	Total mortality rate: 6.5% (39/597) Mortality rate for 29 weeks GA: 14.5% (9 deaths) Mortality rate for 30 weeks GA: 12.9% (15 deaths) Mortality rate for 31 weeks GA: 3.9% (6 deaths) Mortality rate for 32 weeks GA: 3.4% (9 deaths) Early mortality rate (≤ 7 days): 35.89% of total mortality (14/39) Late mortality rate (>7 days): 64.11% of total mortality (25/39)	Lethal congenital and chromosomal anomalies (*n* = 12, 30.77%), sepsis (*n* = 11, 28.20%), severe birth asphyxia (*n* = 6, 15.30%), NEC (*n* = 4, 10.25%), pulmonary hemorrhage (*n* = 3, 7.69%), hydrops/congenital infection (*n* = 3, 7.69%)
Pepler et al. (36)	South Africa	2007–2008	Data for 1,578 infants born in 2007 and 2,376 infants born in 2008 admitted to 15 NICUs in private hospitals	Median GA in 2008: 35.9 weeks (range: 23–42.3 weeks)	2007: 3.1% (49/1,578) 2008: 3.8% (91/2,376)	Not listed
Musooko et al. (37)	Uganda	February 1–March 31, 2013	Data for 635 infants, including 341 infants with severe perinatal morbidity, admitted to a NICU	GA of ≥28 weeks Average GA for the 341 infants: 36.0 ± 1.3 weeks Age at death: 17 (45.9%) of the 37 early neonatal deaths occurred on the first day of life, 7 (18.9%) on the second day, 4 (10.8%) on the third day, and 9 (24.3%) between the fourth and seventh days	Overall mortality rate for all NICU patients in 2012: 26–29% Early neonatal death rate (death within 7 days of birth) of the 635 admissions: 12.4% (79/635) Early neonatal death rate of the 341 infants: 10.9% (37/341)	Not listed

*BPD, bronchopulmonary dysplasia; DIC, disseminated intravascular coagulation; g, grams; GA, gestational age; HMD, hyaline membrane disease; ICH, intracranial hemorrhage; IVH, intraventricular hemorrhage; *n*, number; NEC, necrotizing enterocolitis; NICU, neonatal intensive care unit; RDS, respiratory distress syndrome*.

## Discussion

The age at death for all infants admitted to NICUs globally ranged from 1 to 12 days (20, 22, 25, 33–37). Similarly, deaths in the NICU tended to occur on the first and sixteenth days of life in Israel and Canada, respectively (23, 28). By contrast, very preterm (24) and preterm infants with birth weight of ≤1,500 g (27) were the infants with the oldest age at death. Zhou et al. (24) reported that approximately half [49% (28/58)] of late very preterm infants died in the late neonatal period (7–28 days). Navaei et al. (27) found that the average age at death for preterm infants with birth weight of ≤1,500 g was 28.9 days. It is surprising that very preterm infants had a longer life span than all NICU patients. The delayed mortality in premature infants suggests that a greater percentage of preterm neonates are surviving the immediate conditions of prematurity, only to contract lethal complications later (23). This may be due to the substantial improvements in supportive efforts for these high-risk patients in the first week(s) of life (23). However, it is unclear whether this increased survival for very preterm infants and preterm infants with birth weight of ≤1,500 g is attributable to improved medical care or lengthened suffering prior to death (23).

Consistent with the findings above, studies analyzing the outcomes of all infants admitted to NICUs (20, 22, 25, 33–37) and all in-hospital deaths (23, 28) reported the lowest mortality rates. The mortality rates were 0.2% in Israel (28) and 7.6% in Canada (23), while it ranged from 3.1 to 29% globally (20, 22, 25, 33–37). The mortality rates for the other articles, in ascending order, are as follows: very low birth weight infants [5–44.8%] (30, 32, 39), very preterm infants [8–18.8%] (24, 29), preterm infants (20.6%) (31), and extremely low birth weight infants [34–6%] (21, 26, 27, 38). Among all included analyses, the overall mortality rates for all infants admitted to NICUs and all in-hospital deaths are the lowest, as the study samples include both higher and lower-risk neonates. Conversely, infants who were born premature and with very low birth weight had more severe issues, putting them at a very high risk for death.

The mortality rate of NICUs varies but remains high in both developing and developed countries. Prematurity is a very common etiology of death, as very low birth weight infants (32), infants who died in hospital (23, 28), and preterm infants with very low birth weight (27) were all prematurely born. This could be attributable to the large number of preterm births; in fact, in 2005, the World Health Organization estimated that 12.9 million births of all births (9.6%) in the world were preterm (40).

The findings of this selected review are important for NICUs. Through examining the etiologies of death in different countries, further insight is provided, allowing care providers, policymakers, and researchers to address improvements on areas that will most benefit patients (23). Also, by comparing and contrasting various mortality rates and the age of infants at death, it provides NICUs with many different comparators.

The limitations of our study were that all information was taken from study cohorts. Another weakness was the heterogeneity in the definitions of medical terms (e.g., extremely low birth weight).

## Conflict of Interest Statement

The authors declare that the research was conducted in the absence of any commercial or financial relationships that could be construed as a potential conflict of interest.
